# Supplementary material for: The most uniform distribution of points on the sphere
Source: PLoS One. 2024 Dec 27;19(12):e0313863. doi: 10.1371/journal.pone.0313863 (PMC11676531; doi:10.1371/journal.pone.0313863)
Supplement: S1 Appendix — (PDF) [file pone.0313863.s001.pdf]

Appendix A: The $g(r)$ for random points on a sphere

In this appendix we show how to derive the $P_r(r)$ in the random case, i.e. when the discretisation points are drawn uniformly on the unit sphere. For completeness, we show the computation for generic spin dimension m. The computation is very straightforward: calling $\mathcal{S}_m(1) = 2\pi^{m/2}/\Gamma(m/2)$ the surface of the unit sphere in m dimensions, we write

$$P_r(r) \;=\; \int d\vec{v}_1 d\vec{v}_2 \frac{\delta(v_1-1)}{\mathcal{S}_m(1)}\frac{\delta(v_2-1)}{\mathcal{S}_m(1)}\delta(r-|\vec{v}_1-\vec{v}_2|) = \iint_{\mathbb{S}^2_m} \frac{d\hat{v}_1}{\mathcal{S}_m(1)}\frac{d\hat{v}_2}{\mathcal{S}_m(1)}\delta(r-\sqrt{2(1-\hat{v}_1\cdot\hat{v}_2)}) \tag{A1}$$

$$= \frac{\int_0^\pi \, d\theta\, (\sin\theta)^{m-2}\delta(r-\sqrt{2(1-\cos\theta)})}{\int_0^\pi \, d\theta\, (\sin\theta)^{m-2}} = \frac{\int_0^\pi \, d\theta\, (\sin\theta)^{m-2}\frac{\delta\left(\theta-\arccos\left(1-\frac{r^2}{2}\right)\right)\sqrt{2(1-\cos\theta)}}{\sin\theta}}{\int_0^\pi \, d\theta\, (\sin\theta)^{m-2}}$$

$$= \frac{r^{m-2}(1-\frac{r^2}{4})^{\frac{m-3}{2}}}{A_m}\vartheta(r)\vartheta(2-r) \Longrightarrow \frac{r}{2}\vartheta(r)\vartheta(2-r), \qquad m=3$$

where we set $A_m = \int_0^\pi \, d\theta\, (\sin\theta)^{m-2}$ and $\vartheta(x)$ is Heaviside Theta. The pdf of related quantities such as the scalar product $\hat{v}_1\cdot\hat{v}_2$ (i.e. the latitude of a point) and the angle $\theta = \arccos(\hat{v}_1\cdot\hat{v}_2)$ (i.e. geodesic distance on the unit sphere between two points) can be derived easily in a similar manner.
